# Supplementary material for: Airway and Oral microbiome profiling of SARS-CoV-2 infected asthma and non-asthma cases revealing alterations–A pulmonary microbial investigation
Source: PLoS One. 2023 Aug 17;18(8):e0289891. doi: 10.1371/journal.pone.0289891 (PMC10434894; doi:10.1371/journal.pone.0289891)
Supplement: Attachment — Submitted filename: Response to Reviewers_PONE-D-23-05664R1.docx [file pone.0289891.s002.docx]

**Rebuttal Letter**

Dear Editor,

We were pleased to have an opportunity to revise our manuscript [PONE-D-23-05664R1] "**Airway and Oral Microbiome Profiling of SARS-CoV-2 Infected Asthmatic and Non-Asthmatic Cases Revealing Alterations – A Pulmonary Microbial Investigation**". We have carefully addressed the reviewers' comments and suggestions in the revised manuscript. As instructed, we have attempted to succinctly explain changes made in reaction to all comments. We reply to each comment in a point-by-point fashion. The responses to the concerns raised by reviewers are attached to this letter. The corrections are properly updated in the manuscript, and the changes are highlighted. The reviewers' comments were very helpful overall, and we appreciate such constructive feedback on our original submission. After addressing the issues raised, we feel the quality of the manuscript is much improved. The manuscript has been revised to address the reviewer's comments, which are appended alongside our responses to this letter.

We hope the revised manuscript is accepted for publication in your reputed Journal.

Thank you.

**PONE-D-23-05664R1**

**Reviewer #1:**

The authors present a study analyzing the composition and potential role of the oro-pharyngeal microbiome in the increased susceptibility of asthmatic subjects to COVID19.
The topic is interesting and sound, however I have several major criticisms.
First, the paper is quite difficult to read, due to several shortages in the use of the English language, and should be rewritten eìwith the help of a mother tongue.

1. In particular, there are terms and sentences that must be replaced by correct ones (es: sentence in ines 41-43 of abstract;"incitant" page 2; sentence in lines 74-75 page 3; is instead of are line 120 page 5; between instead of in, line 186 page 7; verbes used in different times throughtou the materials and methods and results sections; etc etc)

Response: As per the reviewer's suggestions the above-mentioned changes have been made to the manuscript and the changes have been highlighted.

2. Second, the abstract should be rewritten to evidence in the corect order: background, aim, methods, results, and conclusions, whereas in the current form has two-third of introduction, one conclusive sentence before results (lines 45-47), and results and methods schematically confined in the last lines (47-55, starting from Findings:).
   Also the rest of the manuscript suffers from the same problems.

Response: The abstract is reordered as suggested by the reviewer. The remaining manuscript sections are modified accordingly.

3. More specifically, in Methods it is difficult to understand if the authors performed "experiment" (as they declare in the abstract) or rather use the already originated metadata (on Asthma study) to analyze existing data aimed to evidence alterations in nasopharyngeal microbiome.

Response: As mentioned in the study, we used the samples from the nasopharyngeal and salivary microbiomes of patients with and without preexisting asthma in response to SARS-COV-2 infection; the BioProject ID: PRJEB51261 has also been mentioned in the manuscript. Further, it has also been mentioned that the dataset files were obtained from the SRA database using the SRA toolbox.

4. The two groups are very disonìmogeneous as to the number (23 vs 82) and this must be justified. Beside, it seems that of the 23 subjects the authors have saliva from 16 and nasopharyngeal samplkes from 8 (and 8 subjects is a really low number to make significant comparisons!)

Response: The number of samples is not defined by the authors. The dataset is made publicly available by the depositors in the SRA under the BioProject ID: PRJEB51261 and is accessed to conduct this experiment. The authors introduce no bias, and all the samples provided in the repository under the particular ID are used for the study.

5. Table 1 of the Methods should be moved to the Results.

Response: As per the reviewer's suggestions the, Table 1 has been moved to the results part.

6. The quality of the Figures is very porr, they are barely readable and the resolution should be increased.

Response: The figure quality is enhanced to 300 DPI. Some images might still have less visibility due to their dense representation.

7. The figure legends should be more explicative, since in the actual form they are very synthetic and do not allow to understand the meaning; the correspondent text in the manuscript should also display more in deep the results and their meaning.

Response: The figure legends are modified per the reviewer's suggestions.

8. The discussion is a summary of the results and should instead discuss the obtained data in light of the previous published data, compareng them and highlitinh similarities and differences.

Response: No detailed study exists in how we analyzed the samples specific to the dataset (i.e. SARS-CoV-2 affected individuals with preexisting asthmatic condition). So, instead of comparing the findings with existing results, we explained the microbiome markers in this study.

9. The paragraph "Machine learning" should be moved to Methods, leaving in the Results section only the data obtained and not the description of the method itself.

Response: As per the reviewer's suggestions, the description part of the "Machine learning" paragraph has been moved to the methods part, and the results are presented in the same section.

10. The conclusion chapter starts with a sentence that has no sense: "demonstrated the significance between"... please rephrase it and try giving a conclusion that put the results in a perspective of importance and possible usage in the treatment of patients.

Response: The conclusion section is modified per the reviewer's suggestions.

11. Also, the bibliography should be updated with the recent papers on the topic (for example, the role of oral microbiome dysbiosis in the COVID19 patients and its relation with severe symptoms was reported in doi: 10.3389/fmicb.2021.687513, and should be included).

Response: As per the reviewer's suggestion, the recent papers on the present study are added to the bibliography.

**Reviewer #2**

**Major Comments:**

1. Although the authors looked at microbial differences between 2 different sample sites, different sexes and different races, which was a strength. However, no conclusions should be made based on this data without baseline microbiomes prior to SARS-CoV-2 infection. The differences between the SARS-CoV-2-Asthmatic and SARS-CoV-2-non-Asthmatic types could be due to the asthma itself, and not the SARS-CoV-2 infection.Thus, I do not believe that their conclusions are supported by their data.

**Minor comments.**

1. Line 70: Change asthmatics to "people with asthma"

Response: As per the reviewer's suggestion, the term has been modified and highlighted.

2. Strengthen the argument on why the microbiome of patients with asthma is affected by SARS-CoV-2.

Response: Immunity has been known to play a very important role in the cause of pathogenesis in asthma. Researchers have postulated that asthma patients may be at high risk of being infected with SARS-CoV-2 and experiencing more severe outcomes when infected. People with asthma harbor an altered airway microbiota linked to increased susceptibility to severe illnesses upon viral respiratory infections. However, the microbiomes of patients with asthma during SARS‐CoV‐2 infection have not yet been characterized properly. Consequently, the current study examines the microbial composition in nasopharyngeal and saliva samples from COVID‐19 patients with and without preexisting asthma.

3. Line 81-83: How does finding elevated expression of ACE2 during COVID-19 infection lead to dysbacteriosis?

Response: SARS-CoV-2 infects human cells by using angiotensin-converting enzyme 2 (ACE2) as a receptor, cleaved by transmembrane proteases during host cell infection, thus reducing its activities. ACE2, a homologue of ACE, has been described as a negative regulator of the Renin-Angiotensin System (RAS), alleviating the deleterious actions mediated by Ang II signaling through Ang II receptor type 1 (AT1R) ([Kuba et al., 2013](https://www.ncbi.nlm.nih.gov/pmc/articles/PMC7365123/#bib0390)). This role is extremely relevant in pathological conditions associated with RAS overactivation, including those related to cardiovascular, renal, and pulmonary systems ([Cole-Jeffrey et al., 2015](https://www.ncbi.nlm.nih.gov/pmc/articles/PMC7365123/#bib0140)). Besides, ACE2 also displays non-RAS-related roles linked with neutral amino acids transport and gut homeostasis; elevated ACE2 expression or function are potentially promoters of intestinal dysbiosis ([Perlot and Penninger, 2013](https://www.ncbi.nlm.nih.gov/pmc/articles/PMC7365123/#bib0525)). This is aligned with the gastrointestinal (GI) symptoms, such as nausea and diarrhea, reported in COVID-19 patients, suggesting an impact on the gastrointestinal-enteric system ([Kotfis and Skonieczna-Zydecka, 2020](https://www.ncbi.nlm.nih.gov/pmc/articles/PMC7365123/#bib0370)).

References:

Kuba, K., Imai, Y., & Penninger, J. M. (2013). Multiple functions of angiotensin-converting enzyme 2 and its relevance in cardiovascular diseases. *Circulation Journal*, *77*(2), 301-308.

Cole-Jeffrey, C. T., Liu, M., Katovich, M. J., Raizada, M. K., & Shenoy, V. (2015). ACE2 and microbiota: emerging targets for cardiopulmonary disease therapy. *Journal of cardiovascular pharmacology*, *66*(6), 540.

Perlot, T., & Penninger, J. M. (2013). ACE2–From the renin–angiotensin system to gut microbiota and malnutrition. *Microbes and infection*, *15*(13), 866-873.

Kotfis, K., & Skonieczna-Żydecka, K. (2020). COVID-19: gastrointestinal symptoms and potential sources of 2019-nCoV transmission. Anaesthesiol Intensive Ther 2020; 40157.

4. Line 318: Was this statistically significant? Were any of the findings statistically significant?

Response: Yes. The statistical significance between the subgroups is explained through alpha, beta diversity analysis, and LDA scores by cladogram analysis.

5. Line 322: The authors indicated that the analysis showed a significant variation yet the p-values are >0.05

Response: The findings reported in the manuscript conclude that there is little significance between the asthmatic and non-asthmatic SARS-CoV-2 infected groups identified in both nasal and oral microbiome samples.

6. Line 350: The authors can't make the conclusion that the abundance of Streptococcus at the genus level in SARS-CoV-2-Asthmatic samples on salivary microbiome strengthens the proof of an impact on the host respiratory condition with microbial dysbiosis. They don't have control samples from the Asthma population before SARS-CoV-2 infection. The dysbiosis could stem from asthma itself, not SARS-CoV-2.

Response: Our conclusion does not emphasize the abundance of Streptococcus as the proof but reports the findings as the discriminating evidence between Asthmatic/non-Asthmatic cases having SARS-CoV-2 condition. It is similar to an example of comparing the high-risk HPV vs low-risk HPV cases to understand the biological differences, thereby could treat with effective medications among the subgroups.

7. In general, grammar should be improved throughout the manuscript.

Response: Based on the reviewer's suggestion, the manuscript's grammar has been improvised.
