# Supplementary material for: Airway and Oral microbiome profiling of SARS-CoV-2 infected asthma and non-asthma cases revealing alterations–A pulmonary microbial investigation
Source: PLoS One. 2023 Aug 17;18(8):e0289891. doi: 10.1371/journal.pone.0289891 (PMC10434894; doi:10.1371/journal.pone.0289891)
Supplement: Attachment — Submitted filename: Response_PONE_D_23_05664_R2.docx [file pone.0289891.s003.docx]

**Reviewer #2:**

**Comment:** Thank you for revising the manuscript. My original concern has not been addressed. You still have not shown the microbial data from uninfected samples. If that is not feasible to do, then please address that in the discussion. Add a "limitations" paragraph where you address some of the limitations of the study such as the low number of some of the sample types as well as the lack of uninfected controls.

**Response:** Based on reviewer suggestion, we have added a separate section as “Limitation of the present study” after the conclusion.
